# Short-Time Recurrences of *Plasmodium vivax* Malaria as a Public Health Proxy for Chloroquine-Resistance Surveillance: A Spatio-Temporal Study in the Brazilian Amazon

**DOI:** 10.3390/ijerph18105061

**Published:** 2021-05-11

**Authors:** Antonio A. S. Balieiro, Andre M. Siqueira, Gisely C. Melo, Wuelton M. Monteiro, Vanderson S. Sampaio, Ivo Mueller, Marcus V. G. Lacerda, Daniel A. M. Villela

**Affiliations:** 1 Instituto Leônidas & Maria Deane, Fundação Oswaldo Cruz (ILMD/Fiocruz), Amazonas 69057-070, Brazil; alcirley@gmail.com (A.A.S.B.); marcuslacerda.br@gmail.com (M.V.G.L.); 2 Programa de Pós-Graduação em Biologia Parasitaria—Instituto Oswaldo Cruz (IOC/Fiocruz), Rio de Janeiro 21040-900, Brazil; 3 Instituto Nacional de Infectologia Evandro Chagas, Fundação Oswaldo Cruz (INI/Fiocruz), Rio de Janeiro 21040-360, Brazil; amsiqueira@gmail.com; 4 Fundação de Medicina Tropical Heitor Vieira Dourado (FMT-HVD), Amazonas 69040-000, Brazil; cardosogisely@gmail.com (G.C.M.); wueltonmm@gmail.com (W.M.M.); vandersons@gmail.com (V.S.S.); 5 Programa de Pós Graduação em Medicina Tropical—Universidade do Estado do Amazonas (UEA), Amazonas 69040-000, Brazil; 6 Fundação de Vigilância em Saúde (FVS), Amazonas 69093-018, Brazil; 7 Walter & Elisa Hall Institute, Melbourne 3052, Australia; mueller@wehi.edu.au; 8 Pasteur Institute, 75015 Paris, France; 9 Programa de Computação Científica, Fundação Oswaldo Cruz (PROCC/Fiocruz), Rio de Janeiro 21040-360, Brazil

**Keywords:** *Plasmodium vivax*, malaria, chloroquine, resistance, disease elimination

## Abstract

In Brazil, malaria caused by *Plasmodium vivax* presents control challenges due to several reasons, among them the increasing possibility of failure of *P. vivax* treatment due to chloroquine-resistance (CQR). Despite limited reports of CQR, more extensive studies on the actual magnitude of resistance are still needed. Short-time recurrences of malaria cases were analyzed in different transmission scenarios over three years (2005, 2010, and 2015), selected according to malaria incidence. Multilevel models (binomial) were used to evaluate association of short-time recurrences with variables such as age. The zero-inflated Poisson scan model (scanZIP) was used to detect spatial clusters of recurrences up to 28 days. Recurrences compose less than 5% of overall infection, being more frequent in the age group under four years. Recurrences slightly increased incidence. No fixed clusters were detected throughout the period, although there are clustering sites, spatially varying over the years. This is the most extensive analysis of short-time recurrences worldwide which addresses the occurrence of *P. vivax* CQR. As an important step forward in malaria elimination, policymakers should focus their efforts on young children, with an eventual shift in the first line of malaria treatment to *P. vivax*.

## 1. Introduction

Malaria caused by *Plasmodium vivax* presents control challenges for several reasons, among them the possibility of increased failure of *P. vivax* treatment. Chloroquine is an antimalarial used as a first-line drug in the treatment of the blood phase of the parasite, but chloroquine resistance (CQR) remains a hurdle. Treatment failure appears as a problem in Brazil and various countries of South America [1,2], Central America [3], and other continents [4,5]. Even with these existing reports of CQR, further studies on the real magnitude of resistance and its space-time distribution are still necessary [6]. Brazil maintains a surveillance system that keeps records of malaria cases (SIVEP-malaria). This system provides individual information on all malaria cases in the Brazilian Amazon basin, allowing data analysis of recurrences as well as incidence of *P. vivax* malaria in the Amazon [7,8,9].

Malaria caused by *P. vivax* differs from *P. falciparum* by the presence of hypnozoites, a latent form present in the liver [10], which may cause relapses, i.e., late episodes of the disease. Recurrences of *P. vivax* malaria consist of three categories: recrudescence, relapse, and reinfection. Recrudescence is the failure to clear asexual blood stages from the circulation, related to ineffective blood schizonticidal treatment. Relapse involves the re-emergence of the disease resulting from the activation of hypnozoites from the liver. Reinfection, in turn, consists of a new infection acquired by the individual through the bite of an infected vector [11].

However, relapse episodes may be mistakenly classified as recrudescence or, in regions of high incidence, as reinfections [12]. Resistance of this species to CQR has been demonstrated in the Brazilian Amazon [6,13,14,15]. According to World Health Organization (WHO), if a drug presents resistance greater than 10%, this drug cannot be used as a first-line drug [12]. The treatment regimen in use in the Amazon region consists of the administration of CQ for three days associated with primaquine (PQ), for seven days, called “short regimen” or for fourteen days, “long regimen” [16].

The National Malaria Control Program (NMCP) of the Brazilian Ministry of Health, classifies recurrences depending on the following times after a first infection: (1) recrudescence, from the 3rd day to the 28th; (2) relapse probable, from 29 to 60 days, and (3) likely reinfection, above 60 days [9].

In order to evaluate the resistance of antimalarial drugs caused by *P. vivax*, clinical trials must be performed [17]. However, we can use information from large datasets such as SIVEP-malaria, which are adjusted using knowledge from clinical trials to construct models and thus make inferences using the observed recurrence time. Recurrence occurs for several reasons [18], including drug resistance, and short-time recurrences are highly linked to recrudescence [3].

In the *P. vivax* malaria literature, there are many reports of CQR, but further large-scale studies on the extent of this resistance are still needed [6]. The analysis of recurrence data up to 28 days, their estimates of trends, and spatial-temporal distribution in epidemiological scenarios in the Brazilian Amazon may reveal important information on the therapeutic failure of CQ. Here, we aim to estimate likely CQR burden, risk factors, trends, and spatial-temporal distribution in distinct epidemiological scenarios in the Brazilian Amazon.

## 2. Methods

### 2.1. Study Area

The study area covers all the Brazilian Amazon from where all malaria notifications are mandatory in the National Malaria Surveillance System (SIVEP-malaria) database of the Health Surveillance Department of the Ministry of Health, active since 2003. The data provided allow surveillance by municipal, state, and federal agencies [19].

### 2.2. Study Design and Data Collection

**Figure 1:**
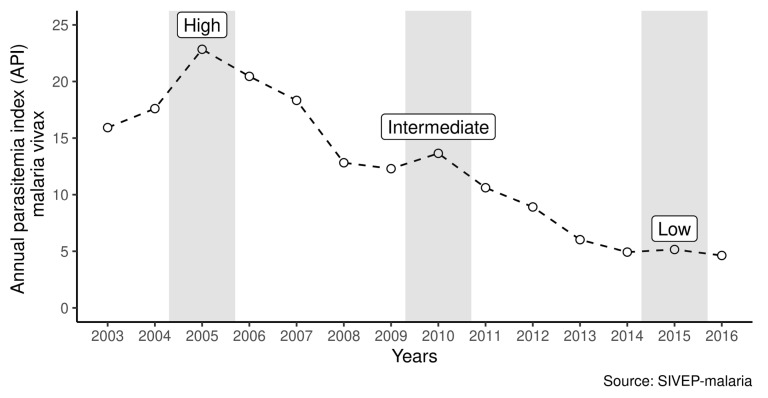
Annual parasitemia index (vivax malaria) during the period from 2003 to 2016 in the Brazilian Amazon.

Datasets containing individual records were used to analyze the risk factors of recurrences for up to 28 days. An observational study design was applied to analyze recurrence risk factors by variables such as age. Spatial analysis as an ecological study also considers data aggregated by municipalities on likely infection in different years, selected according to malaria incidence. Our study focused on three different scenarios, depending on the incidence of *P. vivax* malaria in the Amazon from 2003 to 2016, classified as High (2005), Intermediate (2010), and Low (2015) (Figure 1). In these years, individual-level data provide the number of positive patients for *P. vivax* and thus accounts for the number and time of recurrences. Next, data aggregation of likely infection by municipalities demonstrates the spatial and temporal variation of recurrences for up to 28 days in the Amazon.

### 2.3. Data Processing and Recurrence Identification

Although SIVEP-malaria is a very robust and comprehensive information system, no information on patient follow-up is available. Thus, linking multiple notifications for the same potential patients has some hurdles: the absence of a unique identifier, typos when writing patient names, duplicated entries, and homonymous patients. In the absence of a unique identifier, we use the names of patients along with other personal variables, via linkage tools [9,11]. The linkage to obtain recurrences in the SIVEP-malaria database involved several processes: gathering and organizing the database; standardization of names; searching for similar names; construction of a unique identifier. A probabilistic function using patient’s name, date of birth, mother’s name, federation unit, and municipality of likely infection was applied to identify the same individual in the database. For better computational performance, processing excludes words for joining names and surnames in the patient’s and mother’s names, e.g., accentuation, excess of blanks, numbers, and the prepositions used between the names (“DA”, “DE”, “DAS” e “DOS”). A combination of three components represents the patient by first name, surname, and phonetic name. A soundexBR function returns the phonetic name as an alphanumeric code (soundex code).

We obtained a selection of notification pairs identified as likely to be from the same patients by automatic verification, applying a probability threshold (probability > 0.7). The next step of notification inspection includes visual confirmation of homonyms, possible siblings (twins), and duplicity. A new identification (ID) for notifications from patients deemed as recurrent were provided.

### 2.4. Variables of Interest

The number of days between each individual event of infection was considered the recurrence time within 28 days of infection. Total number of recurrences in the municipality was obtained by counting the number of individuals who were positive from the 5th day to the 28th day, after the first entry in SIVEP-malaria. Recurrence ratio was calculated by dividing the number of recurrences by the number of infections in the municipality. Thus, each municipality that presented records of *P. vivax* malaria in a given year had its percentage of recurrence estimated up to 28 days.

For analysis of the risk factors, we used the individual information on those who recurred up to 28 days, and the comparison group was composed of individuals who had only one entry in SIVEP-malaria. The predictor variables were age (by age group) and the annual parasitemia index (API).

### 2.5. Statistical Analysis

A binomial regression model with random effects in the municipality of likely infection provides the effects of risk factors for recurrence up to 28 days, as odds ratios or predicted probabilities. A multilevel model was applied considering the first level as the individual, and the second level as the municipality.

We used a spatial cluster detection tool with counting data. Because of the study design, some municipalities presented zero recurrences and, in order to deal with that, scan statistics for zero-inflated Poisson (ZIP) models were applied accordingly, which uses a priori information of the expected value of ZIP [20]. The decision to use spatial scanning is due to discontinuous data over time for municipalities.

### 2.6. Software

The SIVEP-malaria databases were imported, and data curated and analyzed in the R software (version 3.5.0), Rstudio Server (version 1.2.1) installed on a server with Linux CentOS operating system version 7, 64 bits, with 90 GB of RAM and 12 physical processing cores. R software packages for larger databases (data.table, RecordLinkage and tidyverse) were used, in addition to GAMLSS, lme4, hnp, soundexBR, scanstatistic, sf, spedp, SpatialEpi and tmap. The significance level for the tests was 0.05.

### 2.7. Ethical Considerations

Access to SIVEP-malaria was duly authorized by the NMPC, respecting the non-disclosure of the identity of patients. The project was submitted to and approved by the CEP-INI/FIOCRUZ (Committee on Research Ethics—Instituto Nacional de Infectologia da Fundação Oswaldo Cruz) (approval number 68577417.2.0000.5262).

## 3. Results

Results were organized to describe the percentages of recurrences up to 28 days at the level of the Brazilian Amazon as a whole, and then its spatio-temporal distribution in the municipalities.

**Figure 2:**
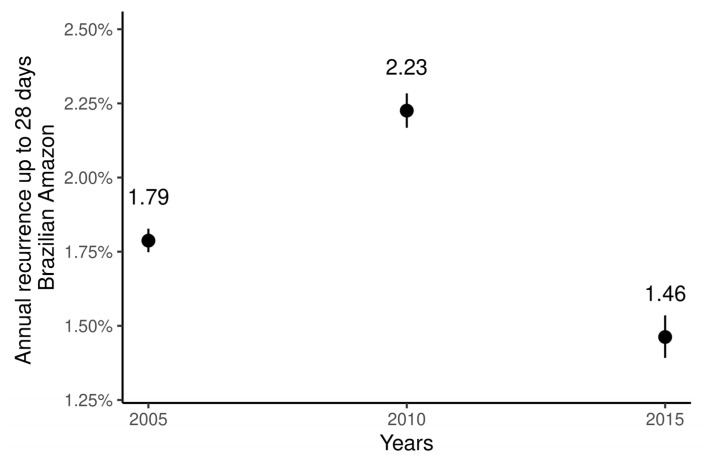
Proportion of short-time recurrences in the Brazilian Amazon in 2005, 2010, and 2015. Bars represent 95% confidence intervals.

The data in Figure 2 represents the overall proportion of recurrences up to 28 days of vivax malaria in the Brazilian Amazon. Both in the high incidence scenario and in others, the percentage has remained below 3%.

**Figure 3:**
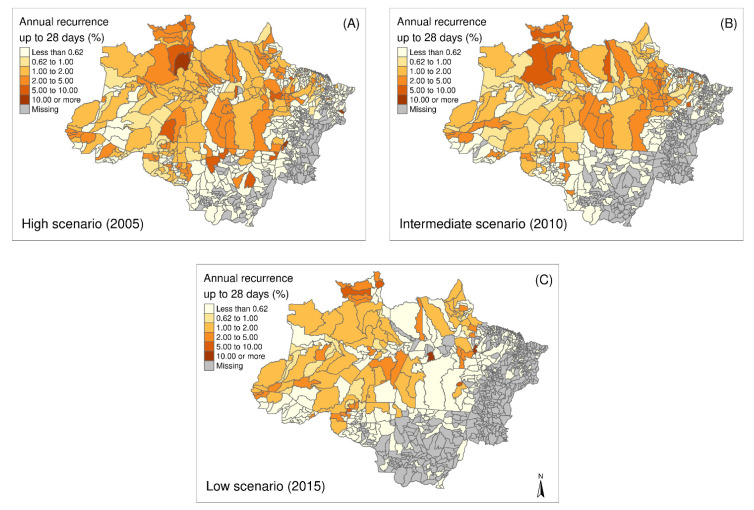
Spatial distribution of percentages of recurrence up to 28 days at the level of municipalities in the Brazilian Amazon in (**A**) 2005–High transmission scenario, (**B**) 2010–Intermediate transmission scenario, and (**C**) 2015–Low transmission scenario.

The three maps represented in Figure 3 describe the scenarios in the studied periods, representing the spatial distribution of the percentage of recurrences for up to 28 days in the Amazon in 2005, 2010, and 2015. The temporal pattern indicates a reduction in the number of municipalities with higher values of recurrence proportion, since for most municipalities values were, in general, less than 5%. Municipalities with rates above 10% were not prevalent. The largest dispersion of municipalities with rates below 5% was found in the state of Amazonas, mainly in the north and at the border with the state of Acre. Proportions of short-time recurrences are concentrated in the range of 1–5% (Figure 3A–C). Most municipalities in 2015 showed the same pattern observed in 2005.

A total of 351/566 (2005) municipalities in the region had zero recurrences up to 28 days, mostly those outside the states of Amazonas and Pará. Over time, the municipalities in these two states, once with more recurrences, experienced a recurrence prevalence decrease. Despite the decline in the number of municipalities with recorded cases of *P. vivax* malaria, recurrence rates in 2015 are not far from those from the peak incidence of *P. vivax* malaria in 2005. Even in the scenario with many *P. vivax* cases, the general pattern of recurrences up to 28 days remained.

**Figure 4:**
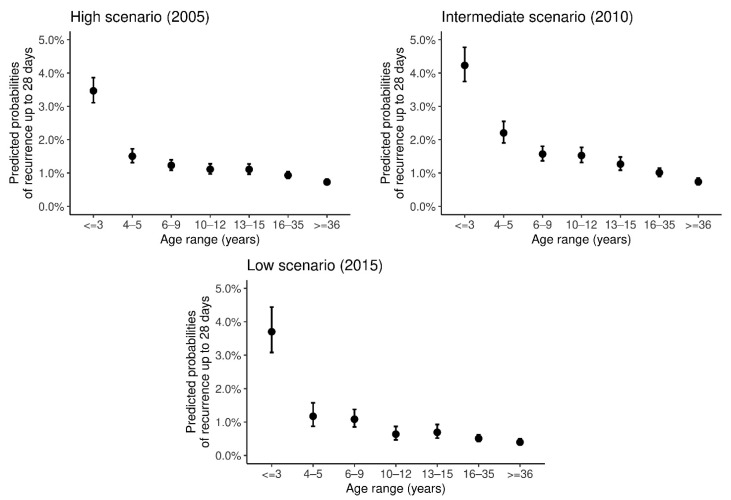
Predicted probability of recurrence up to 28 days according to age group in the Brazilian Amazon in 2005, 2010, and 2015. Bars represent 95% confidence interval for the predicted probabilities.

Using information at the individual level, we list age group (age) as the main risk factor for the probability of recurrence up to 28 days. First, for comparison purposes, we performed the analyzes only with the age group as a risk factor (Figure 4, Table S1). We found that age is the most evident risk factor, especially among children under 4 years old [5,9]. Other factors appearing as significant for a lower risk of recurrence are occupations in hunting/fishing and identification as indigenous persons (Table S2).

The predicted probability of recurrence up to 28 days in ages under four years old is above 3% and below 5%. For this age group, the confidence intervals present intersections between them across the studied years. For the range of 4 to 5-year-olds, and subsequent ages, the probabilities are smaller, and confidence intervals are non-overlapping with that for below four years old. This difference becomes more evident in the year 2010 (Figure 4).

Time to the first recurrence, on average, was close to 28 days in the children under 4 years old group. For the other groups, this average time was apparently higher and this is consistent for the three scenarios evaluated (Figure S1).

**Figure 5:**
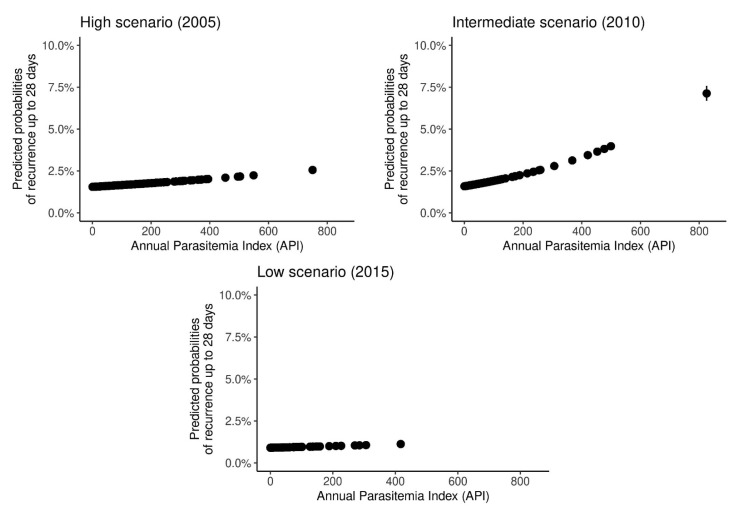
Predicted probability of recurrence up to 28 days according to the annual parasitemia index (API) in the Brazilian Amazon in 2005, 2010, and 2015.

In order to assess the effects of the annual parasitemia index (API) on recurrences, we estimated probabilities of recurrence among municipalities in the three incidence scenarios, demonstrating that the probabilities, regardless of incidence, tend to increase as the API increases (Figure 5). The increasing factor for API was significant, except for year 2015, when incidence was lower (Table S3).

**Figure 6:**
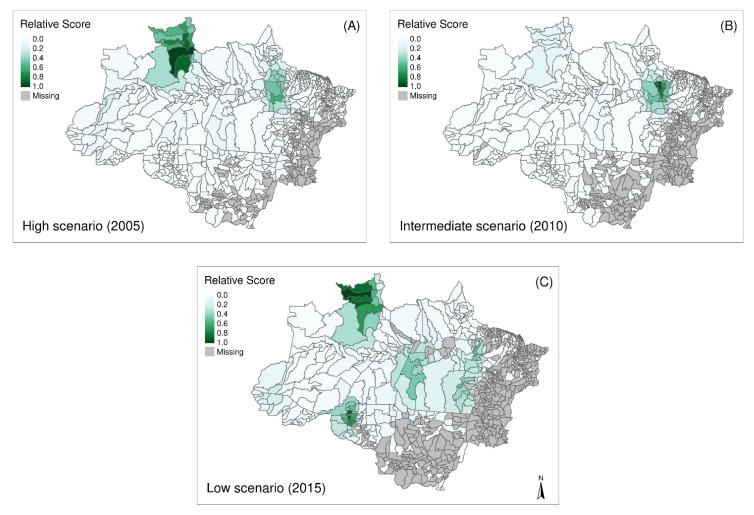
Spatial distribution and detection of clusters of recurrences up to 28 days in municipalities in the Amazon in (**A**) 2005–High transmission scenario, (**B**) 2010–Intermediate transmission scenario, and (**C**) 2015–Low transmission scenario.

Clusters of recurrence up to 28 days were detected in the higher transmission scenario in Roraima (Caracaraí, Rorainópolis, and São Luiz municipalities), Pará (Oeiras do Pará and surrounding), and Amazonas (municipalities on the border of Acre state) (Figure 6A), the first representing that with a higher prevalence of recurrences. In the intermediate scenario (Figure 6B), the clusters from Roraima and Pará remain, but the second becomes the most prevalent. In the low prevalence scenario, the cluster from Roraima again becomes the most prevalent, followed by Pará and Amazonas clusters (Figure 6C).

When comparing the age group related risk of recurrence up to 28 days to recurrences up to 42 days, no significant differences were found (Figure 4 and Figure S2). However, clusters are in different locations, as shown in Figure 6 and Figure S3.

## 4. Discussion

In the three transmission scenarios assessed, recurrence ratios up to 28 days were always estimated below 10% in most of the municipalities. This finding is in agreement with those from clinical trials using the same treatment schemes as in this study [6,14]. Recurrences up to 28 days are expected to prevail when CQ resistance is detected. The low proportion of recurrences found in our study would be attributed to the concomitant use of primaquine, recommended for *P. vivax* treatment in Brazil, except in pregnant women [6,14]. Previous studies have also shown that the use of this combination witnesses decreases in both relapses and recrudescence [5]. Furthermore, the cluster analysis demonstrated a spatial distribution not constant in time, probably related to inefficient control measures or logistical issues rather than parasite genetic characteristics.

Resistance to *P. vivax* was already associated with likely recrudescence, defined as episodes that occur before the 16th day from the first episode, and with likely relapse or recrudescence, when the second episode occurs between the 17th and 28th day after the first [21]. Although less sensitive, defining recurrence up to D28 as associated with CQ resistance is more specific and s the criteria used by WHO [22]. There are two concepts of chloroquine-resistance: clinical and parasitological [22,23]. Cases notified in SIVEP-malaria are supposed to be mostly of clinical chloroquine-resistance, seen by the reappearance of symptoms, thus being more associated with high-grade resistance [24,25].

Malaria elimination relies on a pack of specific components and measures. A comprehensive Information System is one of them. Since 2003, the MoH of Brazil defined SIVEP-malaria as the main information system for malaria surveillance in the Amazon and a huge database has been constructed since [9,19]. However, missing data for some variables hinder risk factor analyzes limited to a subset of variables [19].

The findings presented here demonstrate that the probability of recurrence up to 28 days is associated with the age group. In addition, high incidences of vivax malaria in the municipalities increase the variations in recurrence. Recurrences at low API are mostly composed of resistance. The probability of recurrences in municipalities with a high incidence of vivax malaria possibly increases; however, great uncertainty still remains, since municipalities with extreme incidence make inference harder [26]. The analysis here considered autochthonous cases since information on municipalities of likely infection was used in the Amazon region. Occupations of hunting/fishing appeared with lower risk of recurrence, which may be linked to higher degrees of immunity in some highly exposed groups. Limited information on other socio-demographic variables does not permit further analyzes of other factors.

Recurrence rates for children under 4 years showed strong association with the locality of infection in the Amazon. Earlier studies have demonstrated similar findings [5,9], including slow clearance of parasites in smaller children [16], which corroborates that age changes effect recurrences, particularly in this age group, for whom both CQ and PQ dosages vary as a function of weight and age. Chloroquine administration is particularly complicated for children because of the bitter taste and the size of the pills. As an alternative, mothers are used to fractioning or powdering and mixing it with water or juice in order to make it palatable, which can lead to an incomplete therapeutic dose [27], weakening the effect of these drugs [5]. Thus, since this therapeutic failure is prevalent among children, this could partially explain the higher prevalence of recurrence in this group.

The time to first recurrence was lower in the children under 4 years old group as already described [9]. We also demonstrated that this does not seem to be related to malaria incidence since this average time is similar in the three incidence scenarios evaluated. The recurrence average time close to 28 days in this particular age group, allows us to infer that these episodes are likely to be recrudescence due to therapeutic issues [11].

Furthermore, a more frequent recurrence in children may be linked to developing more symptoms, for example, fever, or even another unrelated infection, mistakenly notified as malaria due to selection bias [28]. By contrast, people develop immunity over time [29,30], so immunity in adults might mask an eventual clinical resistance [31]. An adult with milder disease may have a recurrence because of resistance and not seek health care, thus not being registered in the SIVEP-malaria.

The findings presented here show that children aged less than 3 years old are an important group regarding recurrences related to CQ resistance potentially associated with drug dosing. Decision makers should consider this population carefully in order to achieve malaria elimination. Since the first-line treatment of *P. vivax* malaria currently relies on chloroquine, identifying chloroquine-resistance is essential in efforts towards malaria eradication. Maintaining a surveillance of short-time recurrences as a proxy for CQR is a very important approach toward such a goal.

## Data Availability

To access the database, permission from the National Malaria Control Program (NMCP) of the Brazilian Ministry of Health is required.

## Supplementary Materials

The following are available online at https://www.mdpi.com/article/10.3390/ijerph18105061/s1, Figure S1. Time in days until the first recurrence without the upper limit and truncated to 42 days according to the age group in the Amazon in 2005, 2010, and 2015. Figure S2. Predicted probability of recurrence up to 42 days according to age group in the Amazon in 2005, 2010, and 2015. Figure S3. Spatial distribution and detection of clusters of recurrences up to 42 days in municipalities in the Amazon in (A) 2005–High transmission scenario, (B) 2010–Intermediate transmission scenario, and (C) 2015–Low transmission scenario. Table S1. Factor (age range) reported for recurrence up to 28 days of *P. vivax* in Amazon for the years 2005, 2010 and 2015. Table S2. Analysis of socio-demographic factors in short-time recurrences using SIVEP variables. Table S3. Factor (API) reported for recurrence up to 28 days of *P. vivax* in Amazon for the years 2005, 2010 and 2015.
