# Supplementary material for: Short-Time Recurrences of Plasmodium vivax Malaria as a Public Health Proxy for Chloroquine-Resistance Surveillance: A Spatio-Temporal Study in the Brazilian Amazon
Source: Int J Environ Res Public Health. 2021 May 11;18(10):5061. doi: 10.3390/ijerph18105061 (PMC8150757; doi:10.3390/ijerph18105061)
Supplement: Supplementary file 1 [file ijerph-18-05061-s001.zip › ijerph-1166807-supplementary.pdf]

Short-time recurrences of Plasmodium vivax malaria as a public health proxy for chloroquine-resistance surveillance: a spatio-temporal study in the Brazilian Amazon

Antonio A S Balieiro, Andre M Siqueira, Gisely C Melo, Wuelton M Monteiro, Vanderson S Sampaio, Ivo Mueller, Marcus V G Lacerda, Daniel A M Villela *

Supplementary material

Table S1. Factor (age range) reported for recurrence up to 28 days of *P. vivax* in Amazon for the years 2005, 2010 and 2015.

Years	Predictors	recurrence up to 28 days		Multilevel models (Binomial)		
		No	Yes			
		n (%)	n (%)	OR	OR (95% CI)	p value
2005		**n = 375461**	**n = 6552**			
	(Intercept)			0.04	0.03 – 0.04	**<0.001**
	**Age range (years)**					
	<=3	32568 (95.2)	1635 (4.8)	*reference*		
	4 a 5	19116 (97.9)	410 (2.1)	0.43	0.38 – 0.47	**<0.001**
	6 a 9	35599 (98.3)	631 (1.7)	0.35	0.32 – 0.38	**<0.001**
	10 a 12	26499 (98.4)	424 (1.6)	0.31	0.28 – 0.35	**<0.001**
	13 a 15	25232 (98.4)	406 (1.6)	0.31	0.28 – 0.35	**<0.001**
	16 a 35	150484 (98.6)	2098 (1.4)	0.26	0.24 – 0.28	**<0.001**
	>= 36	85963 (98.9)	948 (1.1)	0.20	0.19 – 0.22	**<0.001**
2010		**n = 207133**	**n = 5218**			
	(Intercept)			0.04	0.04 – 0.05	**<0.001**
	**Age range (years)**					
	<=3	18394 (92.5)	1494 (7.5)	*reference*		
	4 a 5	10595 (96.1)	438 (3.9)	0.51	0.46 – 0.57	**<0.001**
	6 a 9	20284 (97.2)	578 (2.8)	0.36	0.33 – 0.40	**<0.001**
	10 a 12	15603 (97.4)	421 (2.6)	0.35	0.31 – 0.39	**<0.001**
	13 a 15	14746 (97.9)	320 (2.1)	0.29	0.26 – 0.33	**<0.001**
	16 a 35	79391 (98.3)	1372 (1.7)	0.23	0.21 – 0.25	**<0.001**
	>= 36	48120 (98.8)	595 (1.2)	0.17	0.15 – 0.19	**<0.001**
2015		**n = 99556**	**n = 963**			
	(Intercept)			0.04	0.03 – 0.05	**<0.001**
	**Age range (years)**					
	<=3	8354 (96.1)	329 (3.9)	*reference*		
	4 a 5	4889 (98.8)	59 (1.2)	0.31	0.23 – 0.41	**<0.001**
	6 a 9	9735 (98.9)	112 (1.1)	0.29	0.23 – 0.35	**<0.001**
	10 a 12	7527 (99.3)	53 (0.7)	0.17	0.12 – 0.22	**<0.001**
	13 a 15	7940 (99.2)	62 (0.8)	0.18	0.14 – 0.24	**<0.001**
	16 a 35	36637 (99.4)	226 (0.6)	0.13	0.11 – 0.16	**<0.001**
	>= 36	24474 (99.5)	122 (0.5)	0.10	0.08 – 0.13	**<0.001**

Random Effects: σ^2 = 3.29 (2005, 2010 e 2015); τ_{00} 0.40 (2005) 0.41 (2010) e 0.43 (2015) - MUN_INFE; ICC = 0.11 (2005), 0.11 (2010) e 0.12 (2015)

Table S2. Analysis of socio-demographic factors in short-time recurrences using SIVEP variables.

Predictors	Recurrence up to 28 days		Multilevel models (Binomial)		
	No	Yes			
	n (%)	n (%)	OR	OR (95% CI)	p-value
(Intercept)			0.03	0.02 – 0.04	**<0.001**
**Age (Years old)**					
<=3	6610 (95.9)	283 (4.1)		*reference*	
4 - 5	3859 (98.8)	45 (1.2)	0.27	0.20 – 0.37	**<0.001**
6 - 9	7733 (98.8)	94 (1.2)	0.31	0.23 – 0.42	**<0.001**
10 - 12	6092 (99.3)	45 (0.7)	0.20	0.13 – 0.31	**<0.001**
13 - 15	6464 (99.3)	48 (0.7)	0.21	0.14 – 0.31	**<0.001**
16 - 35	29264 (99.4)	178 (0.6)	0.15	0.10 – 0.21	**<0.001**
>= 36	19343 (99.5)	93 (0.5)	0.11	0.08 – 0.16	**<0.001**
**Gender**					
Female	32583 (99)	324 (1)		*reference*	
Male	46782 (99)	462 (1)	1.09	0.95 – 1.27	0.228
**Indigenous**					
No	60982 (99)	612 (1)		*reference*	
Yes	18383 (99.1)	174 (0.9)	0.62	0.49 – 0.79	**<0.001**
**Zone**					
Rural	54468 (99)	551 (1)		*reference*	
Urban	24897 (99.1)	235 (0.9)	1.13	0.95 – 1.35	0.163
**Parasitemia***					
< 200	21948 (99.2)	182 (0.8)		*reference*	
200 - 300	13248 (99.1)	126 (0.9)	1.19	0.94 – 1.50	0.139
301 - 500	16566 (99)	168 (1)	1.13	0.91 – 1.40	0.267
501 - 10000	26061 (98.9)	295 (1.1)	1.04	0.86 – 1.26	0.681
100001 - 100000	1496 (99.1)	14 (0.9)	0.64	0.37 – 1.11	0.109
> 100000	46 (97.9)	1 (2.1)	2.45	0.33 – 18.02	0.379
**Years in school**					
Illiterate	8814 (99.3)	64 (0.7)		*reference*	
Incomplete fundamental studies	41984 (99.3)	308 (0.7)	1.24	0.91 – 1.69	0.171
Complete fundamental studies	4935 (99.4)	30 (0.6)	1.16	0.73 – 1.86	0.529
Incomplete high school	6595 (99.4)	38 (0.6)	1.10	0.70 – 1.71	0.690
Complete high school	6620 (99.4)	41 (0.6)	1.26	0.81 – 1.95	0.310
Incomplete undergraduate	542 (99.8)	1 (0.2)	0.37	0.05 – 2.66	0.326
Complete undergraduate (or higher)	807 (99.1)	7 (0.9)	1.87	0.84 – 4.18	0.125
Not applicable (for children up to 7 years old).	9068 (96.8)	297 (3.2)	1.64	1.19 – 2.27	**0.003**
**Economic activity**					
Domestic	7718 (98.8)	93 (1.2)		*reference*	
Agriculture/livestock	18770 (99.3)	133 (0.7)	0.77	0.58 – 1.02	0.069
Tourism/Traveler	1259 (99.1)	12 (0.9)	0.78	0.42 – 1.43	0.413
Gold/Mining	2279 (98.8)	28 (1.2)	1.35	0.81 – 2.26	0.248
Extractivism	574 (98.8)	7 (1.2)	1.51	0.68 – 3.32	0.308
Fishing/hunting	1938 (99.7)	6 (0.3)	0.42	0.18 – 0.97	**0.043**
Others	46827 (98.9)	507 (1.1)	0.76	0.59 – 0.97	**0.027**

Random Effects: $\sigma^2 = 3.29$; $\tau_{00\ municipalities} = 0.32$; ICC = 0.09; $N_{municipalities} = 263$; N observations 80151;

*Parasites per mm^3

OR: Odds Ratio; **CI**: Confidence Interval

Table S3. Factor (API) reported for recurrence up to 28 days of *P. vivax* in Amazon for the years 2005, 2010 and 2015.

Predictors	recurrence up to 28 days		
	Odds Ratios	*CI*	*P*
**2005**			
(Intercept)	*0.0158*	*0.0152 – 0.0164*	***<0.001***
API	1.0007	1.0005 – 1.0008	**<0.001**
**2010**			
(Intercept)	*0.0161*	*0.0155 – 0.0168*	***<0.001***
API	1.0019	1.0018 – 1.0020	**<0.001**
**2015**			
(Intercept)	*0.0091*	*0.0083 – 0.0101*	***<0.001***
API	1.0005	0.9999 – 1.0012	0.115

CI: Confidence Interval 95%

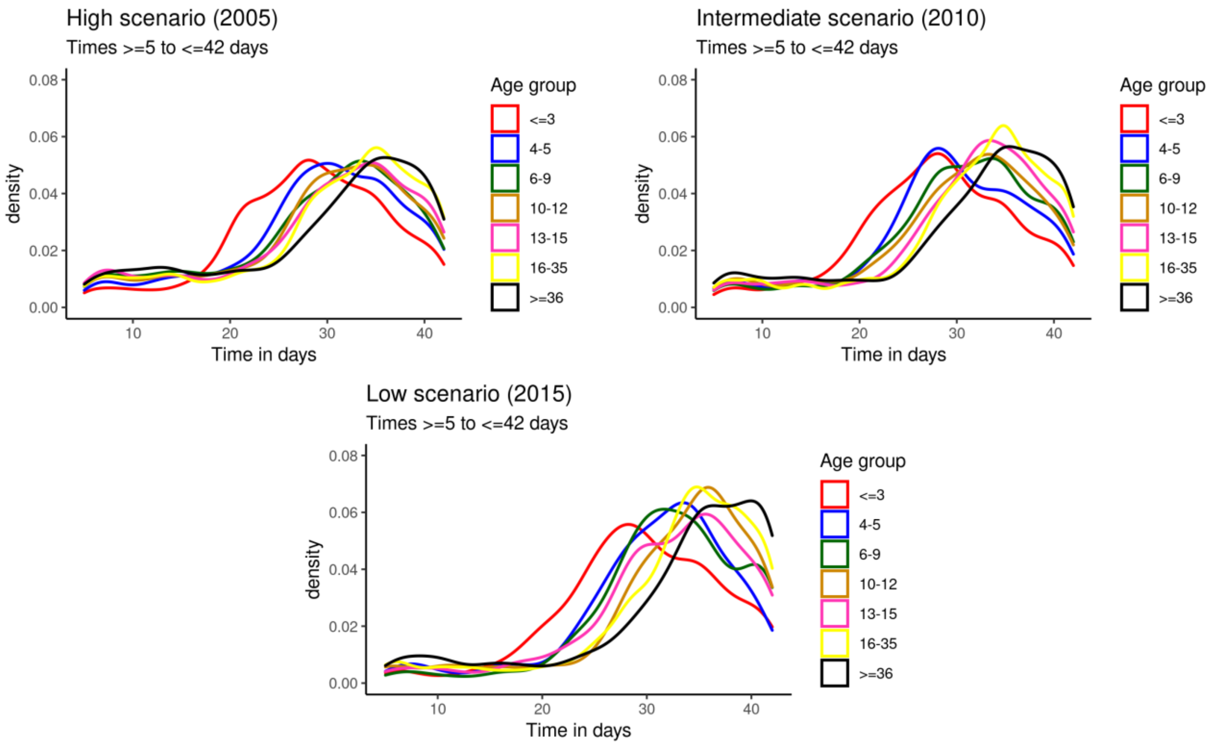

Figure S1. Time in days until the first recurrence without the upper limit and truncated to 42 days according to the age group in the Amazon in 2005, 2010, and 2015.

Result models: Predicted probabilities of recurrence up to 42 days

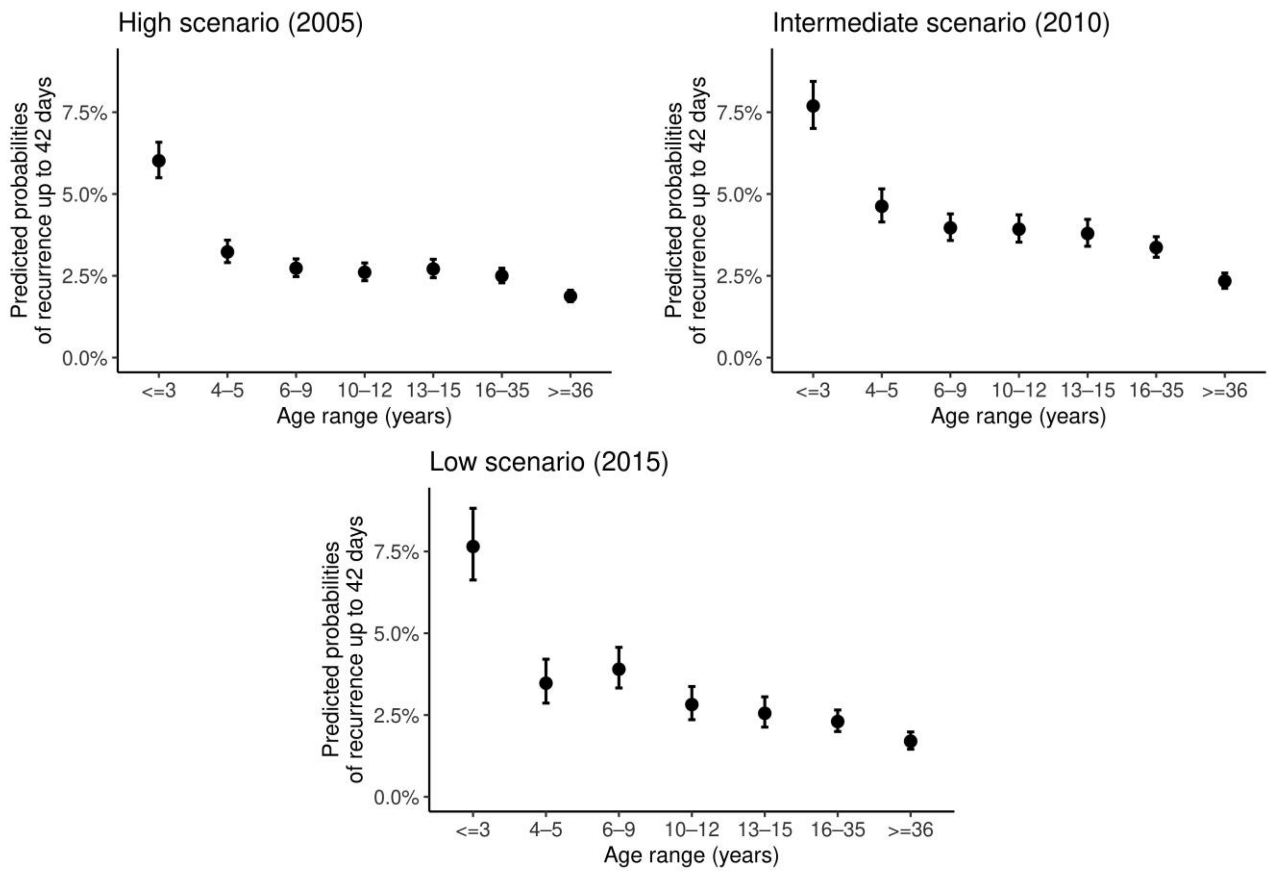

Figure S2. Predicted probability of recurrence up to 42 days according to age group in the Amazon in 2005, 2010, and 2015.

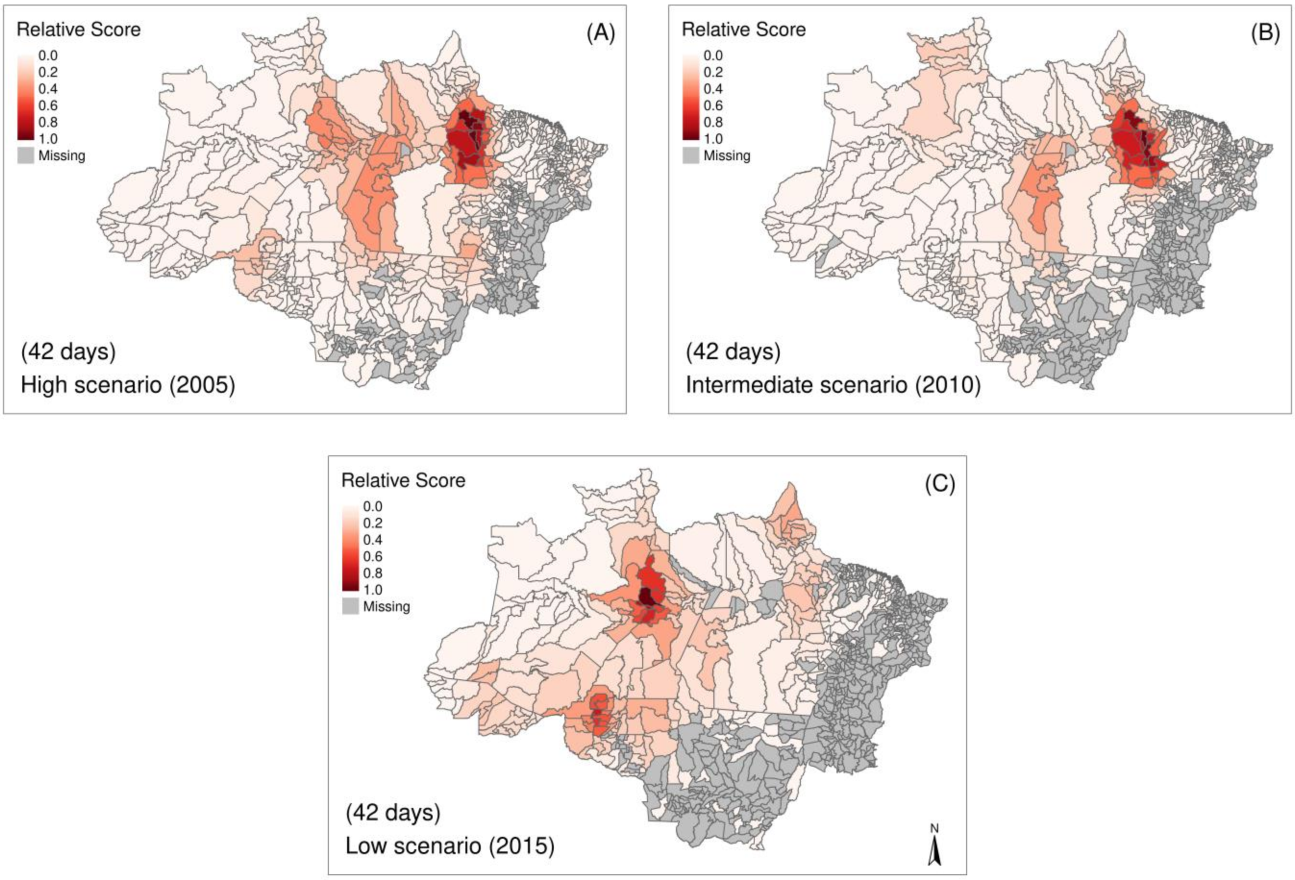

Figure S3. Spatial distribution and detection of clusters of recurrences up to 42 days in municipalities in the Amazon in (A) 2005 – High transmission scenario, (B) 2010 – Intermediate transmission scenario, and (C) 2015 – Low transmission scenario.
